# Association of inflammatory and endothelial cell activation biomarkers with acute kidney injury after sepsis

**DOI:** 10.1186/2193-1801-3-207

**Published:** 2014-04-28

**Authors:** T Clark Powell, Stephen L Powell, Bryant K Allen, Russell L Griffin, David G Warnock, Henry E Wang

**Affiliations:** University of Alabama School of Medicine, Birmingham, Alabama USA; Department of Epidemiology, University of Alabama at Birmingham, Birmingham, AL 35233 USA; Division of Nephrology, University of Alabama School of Medicine, Birmingham, Alabama USA; Department of Medicine, University of Alabama School of Medicine, Birmingham, Alabama USA; Department of Emergency Medicine, University of Alabama School of Medicine, 619 19th Street South, OHB 251, Birmingham, AL 35249 USA; Department of Emergency Medicine, Carolinas Medical Center, Charlotte, North Carolina USA

**Keywords:** Acute kidney injury (AKI), Biomarkers, Endothelium, Inflammation, Sepsis

## Abstract

**Objective:**

Acute kidney injury (AKI) is a sequela of sepsis associated with increased morbidity and mortality. We sought to determine if individuals with elevated baseline levels of inflammation and endothelial cell activation are at increased risk for future AKI after sepsis.

**Methods:**

We conducted an analysis of individuals developing sepsis in the national 30,239 subject REGARDS cohort. Biomarkers measured at the beginning of an 8-year observation period included high-sensitivity C-reactive protein (hsCRP), interleukin-6 (IL-6), tumor necrosis factor (TNF-α), E-selectin, inter-cellular adhesion molecule-1 (ICAM-1), vascular cell adhesion molecule-1 (VCAM-1), and urinary Albumin-to-creatinine ratio (ACR). We defined subsequent sepsis as hospitalization for a serious infection with ≥2 Systemic Inflammatory Response Syndrome (SIRS) criteria. We excluded patients with prior dialysis or kidney transplantation, or those receiving less than two serum creatinine (sCr) measurements during hospitalization. We defined AKI as an increase in sCr ≥0.3 mg/dL from the initial sCr measurement, or the initiation of hemodialysis. Using logistic regression, we evaluated the associations between AKI and biomarker quartiles, adjusting for comorbidities.

**Results:**

We identified 212 sepsis cases encompassing 41 (19.3%) AKI. Elapsed time from biomarker measurement to sepsis episode was 3.1 years (IQR 1.6-4.5). Compared with non-AKI, AKI individuals exhibited higher TNF-α (9.4 vs. 6.2 pg/mL, p = 0.003) and ACR (504.82 vs 61.81 mg/g, p < 0.001). hsCRP, IL-6, E-selectin, ICAM-1 and VCAM-1 were similar between AKI and non-AKI. After adjustment for confounders, AKI after sepsis was more likely in those with higher E-selectin (adjusted ORs 2.91 (0.95-8.93), 1.99 (0.61-6.47), 4.01 (1.30-12.35), test of linear trend p = 0.04), and higher ACR (adjusted ORs 2.29 (0.99-5.30), 10.67 (3.46-32.90), test of linear trend p < 0.001). Baseline hsCRP, TNF-α, IL-6, VCAM-1 and ICAM-1 were not associated with AKI after sepsis.

**Conclusion:**

Elevated baseline levels of E-selectin and ACR are associated with future AKI in the setting of sepsis. Baseline inflammatory and endothelial activation biomarkers may be useful for predicting future risk of AKI in sepsis.

## Background

Acute Kidney Injury (AKI) is the syndrome of rapid loss of kidney function, a process that may result from decreased renal blood flow, obstruction of the outflow tract, or damage to the renal filtration system (KDIGO Clinical Practice Guideline for Acute Kidney Injury. Section 2: AKI Definition 2012). AKI affects over 600,000 patients annually and is associated with over 120,000 deaths (Hoste and Kellum 2006). Sepsis, the syndrome of microbial infection complicated by systemic inflammation, is associated with an estimated 750,000 hospital admissions, 570,000 Emergency Department visits, 200,000 deaths and $16.7 billion in medical expenditures annually in the United States (Angus et al. 2001; Wang et al. 2007). While potentially resulting from shock, surgery and toxic drugs, AKI often ensues following sepsis (Waikar et al. 2008; Bagshaw et al. 2010).

Among the many pathophysiological processes underlying sepsis, inflammation and endothelial activation play prominent roles (Aird 2003; Kellum et al. 2007; Aird 2007). For example, in a cohort of 1,886 subjects hospitalized for community-acquired pneumonia, Kellum, et al. observed that inflammatory cytokines (interleukin-6, interleukin-10, tumor necrosis factor-α) were higher in individuals that developed severe sepsis or septic shock (Kellum et al. 2007). Recent attention has focused on the role of endothelial activation and dysfunction in sepsis pathophysiology (Aird 2003, 2007; Shapiro et al. 2010). In a prior study we found that individuals with high circulating levels of inflammatory and endothelial cell activation biomarkers were prone to future episodes of sepsis (Wang et al. 2013b).

While ample data describe the characteristics of individuals suffering from AKI, there are currently few methods for identifying the individuals at highest future risk for developing AKI. With knowledge of an individual’s risk for developing AKI, it may be possible to initiate treatment at the earliest stages of illness or to introduce measures to prevent AKI. As in sepsis, inflammation and endothelial cell activation are prominent features of AKI pathophysiology. Markers of inflammation and endothelial activation have been associated with AKI in both in vitro and in vivo investigations, especially in the setting of sepsis (Wan et al. 2003; Wan et al. 2008). In this preliminary study, we sought to evaluate the association between such markers of inflammation and endothelial activation at baseline and the risk of future AKI after sepsis.

## Results

Of 300 sepsis hospitalizations, we excluded 85 participants with a history of kidney transplant (n = 8), a history of end-stage renal disease (n = 5), or who received less than two serum sCr measurements during hospitalization (n = 79). Of the remaining 215 cases of sepsis, 41 (19.1%) met criteria for AKI. Elapsed time from biomarker measurement to sepsis episode was 3.1 years (IQR 1.6-4.5). Elapsed times to the sepsis event were similar between AKI (median 2.8 years, IQR: 1.0-4.7) and non-AKI (3.3 years, IQR 1.6-4.5) cases (Wilcoxon rank-sum p = 0.94).

Participants with AKI were more likely to have a history of hypertension, 92% vs 74%; p = 0.03 (Table 1). Among the 41 patients with AKI, most AKI developed within two hospital days (Table 2). All other baseline characteristics were similar between the participant groups. Baseline levels of TNF-α and urine albumin-to-creatinine ratio (ACR) were higher in individuals who developed septic AKI (Table 3).

**Table 1:** **Characteristics of participants hospitalized for sepsis**

Characteristic	AKI	No AKI	p-value*
	(n = 41)	(n = 174)	
Age			
<50	1 (2.4%)	0 (0.0%)	0.21
50–59	3 (7.3%)	26 (14.9%)	
60–69	14 (34.0%)	59 (33.9%)	
70–79	16 (39.0%)	59 (33.9%)	
≥ 80	7 (17.0%)	30 (17.2%)	
Sex			
Male	20 (49.0%)	89 (51.2%)	0.79
Female	21 (51.2%)	85 (48.9%)	
Race			
Black	15 (37.0%)	42 (24.1%)	0.10
White	26 (63.0%)	132 (75.9%)	
Income			
<$20 k	14 (34.0%)	39 (22.4%)	0.29
$20-34 k	12 (29.0%)	46 (26.4%)	
$35-74 k	10 (24.0%)	53 (30.5%)	
≥$75 k	1 (2.4%)	19 (10.9%)	
Refused	4 (9.8%)	17 (9.8%)	
Education			
Less than High School	8 (20.0%)	31 (17.8%)	0.10
High School Grad	10 (24.0%)	40 (23.0%)	
Some College	12 (29.0%)	57 (32.8%)	
College Grad	11 (27.0%)	46 (26.4%)	
Smoking			
Never	15 (37.0%)	53 (30.5%)	0.50
Past	17 (42.0%)	90 (51.7%)	
Current	9 (22.0%)	31 (17.8%)	
Alcohol			
None	23 (56.0%)	110 (63.2%)	0.33
Moderate	15 (37.0%)	54 (31.0%)	
Heavy	1 (2.4%)	8 (4.6%)	
Missing	2 (4.9%)	2 (1.2%)	
Hypertension			
No	3 (7.3%)	45 (25.9%)	0.03
Yes	38 (93.7%)	128 (73.6%)	
Missing	0 (0.0%)	1 (0.6%)	
Coronary Heart Disease			
No	26 (63.0%)	114 (65.5%)	0.74
Yes	15 (37.0%)	58 (33.3%)	
Missing	0 (0.0%)	2 (1.2%)	
Diabetes			
No	23 (56.0%)	116 (66.7%)	0.20
Yes	18 (44.0%)	58 (33.3%)	
Hyperlipidemia			
No	15 (37.0%)	48 (27.6%)	0.39
Yes	26 (63.0%)	123 (70.7%)	
Missing	0 (0.0%)	3 (1.7%)	
Baseline Renal Function (estimated glomerular filtration rate)			
≥90 mL/min per 1.73 m^2^	8 (19.5%)	45 (25.9%)	0.01
60–89.9	13 (31.7%)	84 (48.3%)	
45–59.9	7 (17.1%)	25 (14.4%)	
<45	13 (31.7%)	19 (10.9%)	
Missing	0 (0.01%)	1 (0.6%)	

Excludes individuals with a prior history of kidney transplant or end-stage renal disease, or individuals receiving only one creatinine measurement during hospitalization. *By Chi-square test. AKI = acute kidney injury.

**Table 2:** **Characteristics of creatinine measurements among participants hospitalized with sepsis**

Characteristic	AKI	No AKI
	(n = 41)	(n = 174)
Initial creatinine (mg/dL – median, IQR)	1.8 (1.3-2.8)	1.2 (0.9-1.7)
Maximum creatinine (median, IQR)	2.6 (1.9-4.0)	1.3 (1.0-1.7)
Maximum rise in creatinine from index creatinine (median, IQR)	0.6 (0.4-1.0)	0.0 (0.0-0.1)
Number of measurements to maximum creatinine (median, IQR)	3.0 (2.0-4.0)	1.0 (1.0-2.0)
Elapsed days to maximum creatinine (median, IQR)	1.8 (0.6-2.8)	0.0 (0.0-0.6)

Excludes individuals with a prior history of kidney transplant or end-stage renal disease, or individuals receiving <2 creatinine measurement during hospitalization. AKI = acute kidney injury.

**Table 3:** **Baseline biomarkers and acute kidney injury (AKI) after hospitalization for sepsis**

Biomarker	AKI	No AKI	p-value*
	Median (IQR)	Median (IQR)	
	(n = 41)	(n = 174)	
C-Reactive Protein (mg/dL)	3.23 (1.55-8.60)	3.27 (1.75-6.17)	0.91
TNF-Alpha (pg/mL)	5.22 (4.15-7.76)	7.04 (4.94-9.74)	0.009
IL-6 (pg/mL)	3.48 (2.28-5.60)	3.81 (2.73-6.12)	0.19
E-selectin (ng/mL)	46.15 (33.36-60.17)	49.03 (36.93-66.90)	0.11
VCAM (ng/mL)	1171.17 (1005.17-1343.42)	1186.91 (1055.58-1429.28)	0.38
ICAM (ng/mL)	150.80 (125.50-189.63)	151.10 (117.22-194.74)	1.00
Urine Albumin-to-Creatinine Ratio (mg/g)	9.09 (5.88-24.39)	38.57 (9.57-313.39)	<0.001

*Comparison by Wilcoxon Rank-Sum test.

After adjusting for hypertension and baseline eGFR, the highest quartile of E-selectin was associated with AKI (OR 4.01; 95% CI: 1.27-12.69). (Table 4) We observed a statistically significant linear trend between E-selectin quartiles and the odds ratios for AKI (p = 0.045). The highest tertile of ACR was also associated with the development of sepsis (OR 10.67; 95% CI: 3.46-32.90). We observed a linear trend between ACR tertiles and odds ratios for AKI (p < 0.001). Baseline CRP, TNF-α, IL-6, VCAM and ICAM were not associated with AKI after sepsis.

**Table 4:** **Associations between baseline biomarkers and acute kidney injury (AKI) after hospitalization for sepsis**

Biomarker (Quartiles or tertiles)	Odds ratio (Unadjusted)	Odds ratio (Adjusted for Hypertension and baseline eGFR)	P-trend
C-Reactive Protein			
<1.62 mg/dL	Ref	Ref	0.73
1.62-3.31	1.18 (0.44-3.07)	0.95 (0.34-2.60)	
3.31-7.68	1.35 (0.53-3.48)	1.21 (0.45-3.24)	
>7.68	0.78 (0.28-2.17)	0.74 (0.25-2.13)	
TNF-α			
<4.26 pg/mL	Ref	Ref	0.23
4.26-5.71	0.82 (0.28-2.45)	0.74 (0.24-2.26)	
5.71-8.09	1.13 (0.40-3.18)	0.95 (0.32-2.79)	
>8.09	2.73 (1.06-7.06)	1.68 (0.60-4.71)	
IL-6			
<2.41 pg/mL	Ref	Ref	0.50
2.41-3.56	1.84 (0.66-5.11)	1.60 (0.55-4.65)	
3.56-5.92	1.35 (0.46-3.93)	1.11 (0.36-3.40)	
>5.92	2.20 (0.80-6.07)	1.58 (0.58-4.82)	
E-selectin			
<35.05 ng/mL	Ref	Ref	0.045
35.05-47.09	3.17 (1.04-9.63)	2.70 (0.86-8.46)	
47.09-62.48	2.05 (0.64-6.56)	1.74 (0.52-5.76)	
>62.48	3.68 (1.22-11.12)	4.01 (1.27-12.69)	
VCAM			
<1011.85 ng/mL	Ref	Ref	0.73
1011.85-1172.06	1.16 (0.43-3.14)	1.31 (0.46-3.77)	
1172.06-1352.13	1.05 (0.38-2.89)	0.83 (0.29-2.40)	
>1352.13	1.55 (0.60-3.99)	1.29 (0.46-3.58)	
ICAM			
≤122.38 ng/mL	Ref	Ref	0.97
122.39 = 150-.76	0.55 (0.21-1.49)	0.62 (0.22-1.73)	
150.77-192.26	0.72 (0.28-1.85)	0.70 (0.26-1.89)	
>192.26	0.85 (0.34-2.15)	0.96 (0.37-2.54)	
ACR			
<30 mg/g	Ref	Ref	<0.001
30–300	2.60 (1.14-5.96)	2.13 (0.90-5.02)	
>300	12.73 (4.19-38.66)	9.70 (2.81-33.52)	

Multivariable model adjusted for history of hypertension and estimated glomerular filtration rate (eGFR). Test of trend applied to adjusted model.

## Discussion

Previous studies indicate that biomarkers of inflammation and endothelial cell activation are increased in sepsis, and that sepsis is a major cause of Acute Kidney Injury (AKI) (Chertow et al. 2005; Hoste and Kellum 2006; Angus et al. 2001; Aird 2007). In this preliminary study, we observed associations between baseline biomarkers E-Selectin and urine albumin-to-creatinine ratio (ACR) and the risk of future AKI after sepsis. This association persisted after adjustment for potential confounders. Our observations support the hypothesis that before overt renal cellular injury has occurred, there may be alterations in microcirculation and tissue oxygenation that predispose individuals to renal damage (Okusa et al. 2013). This concept is especially relevant to the septic state, where inflammation and endothelial cell activation are prominent (Cheng et al. 2012).

There are plausible connections between inflammation and endothelial cell activation and acute kidney injury. TNF-α is an inflammatory marker released by activated macrophages, monocytes and neutrophils and has been shown to have a major role in both sepsis and septic AKI (Aird 2007; Wolfs et al. 2002; Xu et al. 2013; Wan et al. 2003). Renal endothelial cells are activated by TNF-α, further perpetuating the pro-inflammatory state and potentially sensitizing kidney tissue to subsequent damage (Aird 2007; Wolfs et al. 2002). Acute endothelial cell changes may lead to altered vascular reactivity, permeability, adherence of leukocytes, coagulation and microvascular vasomotor autoregulation, perpetuating AKI.

Various studies have shown albuminuria to be a result of endothelial activation, capillary permeability, and systemic inflammation in both the baseline and septic states (Zhang et al. 2012). Albuminuria is established as an indicator of kidney function in the general population and has been shown to predict AKI, length of stay, and mortality in the acute care setting (Zhang et al. 2012; Gansevoort et al. 2011). However, our approach is novel as there have been no evaluations of the association of baseline ACR measured prior to hospitalization for sepsis with risk of future AKI development.

The novelty of our study is that the indicators of abnormal inflammatory and endothelial cell response were detected at baseline, prior to the onset of sepsis and resulting AKI. These observations highlight the potential use of baseline biomarker profiles to identify individuals at risk of developing AKI, setting the stage for mitigating or preventing AKI in the context of sepsis. For example, in murine models, Coldeway et al. and Choi et al. among others have demonstrated the use of erythropoietin and glucocorticoids as prophylaxis for AKI in sepsis (Choi et al. 2013; Coldewey et al. 2013). While not used in current clinical practice, the identification of individuals at risk for future AKI has tremendous implications for care. This information could guide clinicians in the care of individuals at heightened risk for AKI; for example, selecting renal-sparing medications, avoiding of radiologic contrast, or implementing of renal-protective strategies early in the course of hospitalization. We emphasize that these biomarkers were measured at healthy baseline prior to illness – *not* after the onset of sepsis. Further investigation of inflammatory markers in disease progression is warranted given the robust opportunities for illness prevention and outcomes improvement.

While we focused on select markers of inflammation and endothelial cell activation, other studies have identified markers associated with early active AKI. For example, elevated serum and urine levels of Neutrophil Gelatinase-Associated Lipocalin (NGAL), a glycoprotein produced by epithelial tissues throughout the body, have been associated with AKI in the setting of sepsis (Ostermann et al. 2012; Bagshaw et al. 2010; Martensson et al. 2010). Kidney Injury Molecule-1, a transmembrane glycoprotein produced by damaged proximal tubular cells is a marker of early AKI (Ostermann et al. 2012; Han et al. 2008; Liangos et al. 2009). Interleukin-18, a pro-inflammatory cytokine released from injured proximal tubule cells, has been used in conjunction with glomerular filtration rate diagnose or predict progression of AKI (Liangos et al. 2009; Endre et al. 2011). Other markers of AKI include Cystatin C and Liver-type Fatty-acid Binding Protein. (Ostermann et al. 2012; Herget-Rosenthal et al. 2004; Iannitti et al. 2011). Additional study must identify if these markers can be used to predict the future risk of AKI.

## Limitations

We studied a narrow selection of biomarkers and included a portion of the full REGARDS cohort. We did not consider other potentially relevant biomarkers such as interleukin-10, soluble fms-like tyrosine kinase-1 (sFlt-1), plasminogen activator inhibitor-1 (PAI-1) or NGAL. An ideal study would encompass examination of a broader range of baseline biomarkers involving all subjects in the cohort. However, our preliminary study lays the foundation for a more comprehensive examination of a broader range of biomarkers and mechanisms. Our study examined only a single measurement of each biomarker at the beginning of the REGARDS study. Repeat blood and serum samples are not yet available for REGARDS participants, although a repeat examination of subjects in the cohort is in progress.

We included only individuals receiving two or more creatinine measurements during hospitalization. As is well known, AKI may have occurred but not been detected on individuals receiving fewer sCr measurements. While REGARDS measured baseline sCr on all individuals, from our practical experience there can be considerable drift in sCr values over time, hence making characterization of the true baseline uncertain.

While the prevalence of comorbidities such as hypertension, diabetes and hyperlipidemia seem high in this study, they are in fact similar to the figures observed among sepsis individuals in the larger REGARDS cohort. Because we relied on participant reports of hospitalizations, recall or reporting biases may have resulted in under-identification of sepsis events. However, our approach utilized prospective, systematic, dual review of hospital records using consensus definitions. While we did not examine more advanced sepsis levels such as severe sepsis and septic shock, our study is relevant to these more advanced stages because it identifies individuals at the earliest stages of disease. We also did not study the hospital course of sepsis or repeat sepsis events. Due to the limited available sample size, we did not examine other potential confounders such as medication use.

## Conclusions

In this pilot analysis, elevated baseline E-selectin and ACR were associated with future episodes of AKI after sepsis. Inflammatory and endothelial cell activation biomarkers may play a role in the prediction of AKI risk.

## Methods

### Study design

The Institutional Review Board of the University of Alabama at Birmingham approved the study. We used data from Reasons for Geographic and Racial Disparities in Stroke (REGARDS), a population-based longitudinal cohort. We previously performed a nested analysis using a random sample of sepsis cases from the REGARDS cohort(Wang et al. 2013b).

### The REGARDS cohort

REGARDS is a national community-based cohort that includes 30,239 Black and White adults, age ≥ 45 years old from all regions of the continental United States. The cohort was designed to gather data and identify risk factors for increased incidence of stroke mortality in Blacks and southerners (Howard et al. 2005). The demographic composition of the cohort is: 41% African Americans, 59% Whites, 45% men and 55% women, and 69% individuals over 60 years old. REGARDS enrolled patients from 2003–2007, conducting home visits and utilizing structured interviews to gather baseline information about participants.

Baseline data obtained for each participant included physical characteristics (height, weight), physiology (blood pressure, pulse, electrocardiogram), diet, family history, psychosocial factors and prior residences, as well as biological specimens (blood, urine, etc.). The study contacted each participant on a semi-annual basis to determine the date, location and reason for all emergency department visits and hospitalizations during the prior 6 months. Upon a participant’s death, the study interviewed proxies to ascertain the circumstances surrounding the death.

### Selection of subjects

We identified all hospitalizations for a serious infection during February 5, 2003 – October 14, 2011. We defined serious infection hospitalization based upon taxonomies by Angus, et al (Angus et al. 2001). Two trained abstractors independently reviewed medical records relevant to the infection event, confirming 1) the presence of serious infection on hospital presentation, and 2) that the infection was a primary reason for hospitalization. The abstractors also collected clinical and laboratory information for the first 28-hours of hospitalization. The abstractors resolved all data discordances by consensus review. We provided additional physician-level adjudication as needed. Based upon a review of 1,349 hospital records, inter-rater agreement were strong for the presence of serious infection (kappa = 0.92) and sepsis (kappa = 0.90).

Consistent with international consensus criteria, we defined sepsis cases as hospitalizations for serious infection with two or more systemic inflammatory response syndrome (SIRS) criteria. SIRS criteria included body temperature > 38°C (100.4°F) or < 36°C (96.8°F), heart rate > 90 beats per minute, respiratory rate > 20 breaths per minute or arterial carbon dioxide tension (PaCO2) < 32 mm Hg, and abnormal white blood cell count (>12,000/μL or < 4,000/μL or >10% immature band forms) (Cannell et al. 2006). We did not include end organ dysfunction in the definition of sepsis. We did not include sepsis that developed later in hospitalization.

### Outcomes – definition of acute kidney injury

A range of approaches exist for defining AKI, including the Risk-Injury-Failure-Loss-End Stage Renal Disease (RIFLE) and Acute Kidney Injury Network/Kidney Disease Improving Global Outcomes (AKIN/KDIGO) criteria (Naranbat et al. 2009). More recently, we have proposed an AKI staging system based purely upon absolute changes in sCr (Wang et al. 2013a). Consistent with these consensus criteria, we defined AKI as a sCr increase ≥0.3 mg/dL from the first sCr value during hospitalization, or the initiation of hemodialysis. We did not constrain the sCr rise to a 48 hour window, as in the RIFLE and AKIN/KDIGO criteria. We included only subjects with at least two serum creatinine measurements during hospitalization. We excluded patients with prior dialysis or kidney transplantation. Trained abstractors conducted a detailed chart review to identify all sCr measurements during hospitalization as well as the need for dialysis.

### Selection and determination of biomarkers

Because of the pilot nature of this study, we focused on markers of inflammation and endothelial cell activation with biologically plausible connections to sepsis and AKI pathophysiology. Additional considerations included the sample volume requirement, and the availability and accuracy of serum assays after long-term storage. Our final selection of biomarkers included baseline interleukin-6 (IL-6), tumor necrosis factor (TNF-α), soluble endothelium selectin (E-Selectin – also known as endothelial-leukocyte adhesion molecule 1 (ELAM)), inter-cellular adhesion molecule-1 (ICAM-1), vascular cell adhesion molecule-1 (VCAM-1), and urinary Albumin-to-creatinine ratio (ACR). The REGARDS study also measured high-sensitivity C-Reactive Protein (hsCRP) on all individuals.

Trained personnel obtained all biomarker samples at the time of the participant’s enrollment in to the REGARDS cohort. The study collected blood samples from all REGARDS participants at participants’ homes following a 10–12 hour fast. Study personnel centrifuged and separated serum from plasma within 2 hours of collection (mean 97 minutes, SD 127 minutes). Overnight shipping of samples on ice to the central laboratory was achieved for 95% of the cohort. On arrival at the central laboratory, the samples were centrifuged at 30,000 g and 4°C, and either analyzed or stored at −80°C.

Commercial biomarker kits used in the study included HS ELISA kit (for IL-6 - R&D Systems, Minneapolis, Minnesota), Millipore Singleplex system (for TNF-α - Millipore, Billerica, Massachusetts, USA) and Millipore multiplex assays (for E-Selectin, ICAM-1 and VCAM-1 - Cardiovascular Panel 1, Millipore, Billerica, Massachusetts, USA). hsCRP was determined in batches by particle-enhanced immunonephelometry using the BNII nephelometer (N High-sensitivity CRP; Dade Behring, Deerfield, Illinois) with interassay CVs of 2.1%–5.7%. Urinary albumin and creatinine were measured using the BN ProSpec Nephelometer (Dade Behring, Deerfield, Illinois). The laboratories performed all assays in duplicate, using the average value in the analysis.

All analyses were conducted by The Laboratory for Clinical Biochemistry Research, University of Vermont, Burlington, Vermont, and the Department of Laboratory Medicine and Pathology University of Minnesota, Minneapolis, Minnesota.

### Covariates

Covariates included demographics (age, sex, race, education, income), health behaviors (tobacco and alcohol use) and chronic medical conditions (hypertension, coronary heart disease, diabetes, dyslipidemia). Racial categories include white and black; REGARDS did not recruit subjects from other racial groups. Education categories included less than high school, high school graduate, some college, and college graduate. Income levels included < $20,000/year, $20,000-34,000/year, $35,000-74,000/year, and ≥75,000/year. We also included a category for instances where the participant declined to provide income information.

We defined tobacco use as current, past and never. We defined alcohol use according to the National Institute on Alcohol Abuse and Alcoholism classification: i.e., moderate (1 drink per day for women or 2 drinks per day for men) and heavy alcohol use (>1 drink per day for women and >2 drinks per day for men).

We defined hypertension as baseline systolic blood pressure ≥140 mm Hg, diastolic blood pressure ≥90 mm Hg, or current use of antihypertensive agents. Heart disease included self-reported history of myocardial infarction, coronary artery bypass grafting, or cardiac angioplasty or stenting, or baseline electrocardiographic evidence of myocardial infarction. Diabetes consisted of fasting glucose ≥126 mg/dL, non-fasting glucose ≥200 mg/dL, or the use of insulin or oral hypoglycemic agents. Dyslipidemia included self-reported high cholesterol or ongoing use of lipid lowering medications. We calculated each individual’s estimated glomerular filtration rate (eGFR) based upon sCr measured upon enrollment in REGARDS(Levey et al. 1999).

### Data analysis

We limited the analysis to sepsis cases only. We excluded individuals with a history of kidney transplant or end stage renal disease, or who received less than two sCr measurements during hospitalization. We compared subject characteristics between those who did and did not develop AKI during hospitalization, using the chi-square test for categorical variables and the Wilcoxon Rank-Sum test for continuous variables. Using logistic regression, we evaluated the associations between AKI and quartiles of each biomarker. We also conducted a test of linear trend between the quartiles of each biomarker (Szklo 2014). ACR was divided into three categories consistent with KDIGO nomenclature for staging chronic kidney disease. For multivariable adjustment, we included participant characteristics found to be statistically significant on univariable analysis. We conducted all analyses using Stata Version 12.1 (Stata Corp, College Station, Texas, USA).

## Abbreviations

ACR: Albumin-to-creatinine ratio
AKI: Acute kidney injury
AKIN: Acute kidney injury network
hsCRP: High sensitivity C-reactive protein
ICAM-1: Inter-cellular adhesion molecule-1
IL-6: Interleukin-6
KDIGO: Kidney disease improving global outcomes
NGAL: Neutrophil gelatinase-associated lipocalin
REGARDS: Reasons for geographic and racial differences in stroke
RIFLE: Risk-injury-failure-loss-end stage renal disease
sCr: serum creatinine
sFlt-1: soluble fms-like tyrosine kinase-1
PAI-1: Plasminogen activator inhibitor-1
SIRS: Systemic Inflammatory response syndrome
TNF-α: Tumor necrosis factor
VCAM-1: Vascular cell adhesion molecule-1.
